# Paternity after vasectomy with two previous semen analyses without spermatozoa

**DOI:** 10.1590/S1516-31802007000200011

**Published:** 2007-03-04

**Authors:** Marcos Lucon, Antonio Marmo Lucon, Fabio Firmbach Pasqualoto, Miguel Srougi

**Keywords:** Vasectomy, Infertility, Paternity, Azoospermia, Fertility, Vasectomia, Infertilidade, Paternidade, Oligospermia, Fertilidade

## Abstract

**CONTEXT:**

The risk of paternity after vasectomy is rare but still exists. Overall failure to achieve sterility after vasectomy occurs in 0.2 to 5.3% of patients due to technical failure or recanalization. The objective of this report was to describe a rare but notable case of proven paternity in which the semen analyses had not given evidence of spermatozoa.

**CASE REPORT:**

A 44-year-old vasectomized man whose semen analyses had shown azoospermia became a father four years after sterilization. Blood sample DNA analysis on the child and husband proved biological paternity.

Vasectomy may fail in the long run even without spermatozoa in semen analysis. The patient must be aware of this possibility.

## RESUMO

**CONTEXTO:**

O risco de paternidade após vasectomia é raro, mas existente. Falha em atingir esterilidade após vasectomia ocorre em 0.2% a 5.3% dos pacientes devido à falha técnica ou recanalização. O objetivo é descrever um caso raro, mas importante, de paternidade comprovada cujo espermograma mostrava ausência de espermatozóides.

**RELATO DE CASO:**

Um homem de 44 anos vasectomizado, cujo espermograma evidenciou azoospermia, tornou-se pai após quatro anos da esterilização. Análise sanguínea por DNA da criança e do marido provou paternidade biológica. Vasectomia pode falhar a longo prazo até com azoospermia no espermograma. O paciente deve estar informado dessa possibilidade.

## INTRODUCTION

The risk of paternity after vasectomy is rare but still exists. Overall failure to achieve sterility after vasectomy occurs in 0.2 to 5.3% of patients due to technical failure or recanalization. Early recanalization occurs in 0.36% and delayed recanalization in 1 in 2000.^1^ In these cases, semen analyses give evidence of spermatozoa. When there is postoperative azoospermia, it is believed that sterility is assured. However, six cases of proven paternity of a father without spermatozoa in semen analyses after vasectomy have been reported.^2^

## OBJECTIVE

To describe another case of proven paternity of a father with negative semen analyses after vasectomy.

## CASE REPORT

**Figure 1:**
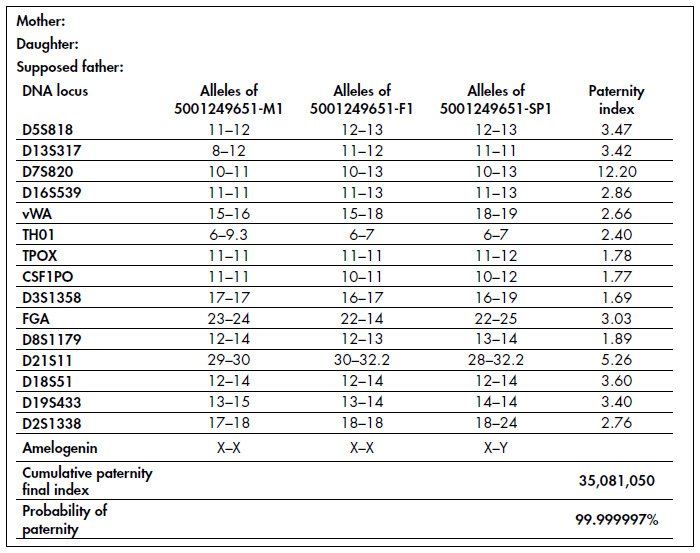
DNA analysis test proving the paternity of a vasectomized father.

A 44-year-old man came to the Department of Urology, Hospital das Clínicas, Faculdade de Medicina da Universidade de São Paulo (FMUSP). He had been vasectomized four years before, and semen analysis three months later had shown azoospermia. Four years after this surgery, his wife conceived and examination of semen one month after the beginning of the pregnancy did not give evidence of spermatozoa. After the delivery of a healthy child, a third sperm analysis showed no spermatozoa. However, blood sample DNA analysis on the child and husband proved biological paternity (Figure 1).

## DISCUSSION

Vasectomy is safe, easy and the most highly cost-effective method of contraception. It generally does not have any sequelae. The most serious complication is scrotal hematoma and, in the majority of cases, this can be treated conservatively.^1,2^

Failure of vasectomy may be a cause for legal action. Although a single negative semen examination indicates that the vasectomy was a success, routine analysis in the United Kingdom calls for two examinations.^3^ In the United States, 56% of the doctors require one, 39% require two and 5% ask for three semen examinations after vasectomy in order to confirm azoospermia.^2^ The risk of delayed recanalization is small and has been estimated to be as low as one in 2000 to 7000 patients.^1^ Late recanalization, shown by the presence of spermatozoa in the semen analysis after a previous negative result, has been found in 0.6 to 1%, although without resulting in pregnancy.^3^

Fertilization after successful vasectomy has already been reported. Smith et al. reported six cases of DNA-confirmed paternity in which semen analysis had been negative before conception.^3^ The case described here resulted in conception four years after vasectomy. The interval between vasectomy and late fertilization has ranged from eight months to ten years in the cases reported.^1-4^ Since fertilization occurred without positive semen analysis, it may be speculated that vas deferens permeability for spermatozoa was intermittent, as has already been suggested.^3^ The resulting risk of conception must not be underestimated. As there is no reliable method for identifying patients who may present recanalization, it is the doctor's responsibility to inform the patient of the remote possibility of fertilization in the long run. It must be emphasized, therefore, that vasectomy is not universally successful. Abstinence remains the only infallible method for contraception.

Therefore, vasectomized patients whose partners become pregnant should be counseled to undergo sperm examination and DNA analysis before any doubt regarding paternity is expressed.^1,4^

## CONCLUSION

Vasectomy may fail in the long run even without spermatozoa in semen analysis after surgery. The patient must be aware of this possibility.
